# Pembrolizumab Every 6 Weeks Versus Every 3 Weeks in Advanced Non-Small Cell Lung Cancer

**DOI:** 10.1093/oncolo/oyad182

**Published:** 2023-06-26

**Authors:** Maude Dubé-Pelletier, Catherine Labbé, Jimmy Côté, Audrey-Ann Pelletier-St-Pierre

**Affiliations:** Institut universitaire de cardiologie et de pneumologie de Québec, Université Laval, Québec, Canada; Institut universitaire de cardiologie et de pneumologie de Québec, Université Laval, Québec, Canada; Institut universitaire de cardiologie et de pneumologie de Québec, Université Laval, Québec, Canada; Institut universitaire de cardiologie et de pneumologie de Québec, Université Laval, Québec, Canada

**Keywords:** advanced non-small cell lung cancer, pembrolizumab 4 mg/kg every 6 weeks, pembrolizumab 2 mg/kg every 3 weeks

## Abstract

**Background:**

The survival benefits and adverse effects of pembrolizumab 2 mg/kg intravenously (IV) every 3 weeks (Q3W) in advanced non-small lung cancer (NSCLC) are well documented in the literature. Based on pharmacokinetic models, a pembrolizumab 4 mg/kg IV every 6 weeks (Q6W) dosing regimen is also approved in some countries. To date, there is no direct comparison in the literature between these 2 regimens in advanced NSCLC.

**Methods:**

This retrospective study included 80 patients with advanced NSCLC who received pembrolizumab monotherapy 4 mg/kg Q6W between March 1, 2020 and December 31, 2021 and 80 patients with advanced NSCLC who received pembrolizumab monotherapy 2 mg/kg Q3W between January 1, 2017 and January 15, 2019 at *Institut universitaire de cardiologie et de pneumologie de Québec* (IUCPQ). The primary outcomes of this study were to compare overall survival (OS), progression-free survival (PFS) as well as the occurrence and severity of immune-mediated adverse events (AEs) in patients with advanced NSCLC who received pembrolizumab Q6W vs Q3W. Data cutoff date was December 15, 2022.

**Results:**

Median follow-up was 14.5 ± 8.6 months in the Q6W group and 18.3 ± 19.6 months in the Q3W group. Median PFS was 6.9 months (CI 95% 5.0-10.7) in the Q6W group vs 8.9 months (CI 95% 5.6-14.1) in the Q3W group (adjusted HR 1.27 (CI 95% 0.85-1.89), *P* = .25). Median OS was not reached in the Q6W group vs 20.5 months (CI 95% 13.7-29.8) in the Q3W group (adjusted HR 0.80 (CI 95% 0.50-1.29), *P* = .36). Immune-mediated AEs of grade ≥ 3 occurred in 18% of patients in the Q6W group and in 19% of those in the Q3W group.

**Conclusions:**

In this unicentric retrospective study, the pembrolizumab Q6W dosing regimen was comparable to the Q3W in terms of OS, PFS, and toxicity.

## Teaser

This study compared overall survival, progression-free survival and immune-related adverse events in patients with advanced non-small cell lung cancer who received pembrolizumab every 6 weeks versus every 3 weeks.

**Implications for Practice**

By demonstrating that the pembrolizumab every 6 weeks is as safe, in terms of efficacy and toxicity, as the 3-weekly dosing regimen, this finding could significantly improve the quality of life of patients with advanced non-small cell lung cancer and reduce health system costs.

## Introduction

Immunotherapy is now well integrated in the treatment algorithm for patients with advanced non-small cell lung cancer (NSCLC). The anti-programmed cell death protein 1 (PD-1) antibody pembrolizumab monotherapy is used for first-line treatment of advanced NSCLC in patients with a tumor proportion score (TPS) for programmed death ligand 1 (PD-L1) of 50% or greater. This treatment is also indicated in first line in combination with chemotherapy for 4 cycles, regardless of PD-L1 status. Thereafter, pembrolizumab is continued for maintenance alone or in combination with chemotherapy, depending on histology. This immune checkpoint inhibitor is also approved as second-line therapy for patients with PD-L1 TPS ≥ 1%, an indication which is less used now that all patients have access to first-line immunotherapy as monotherapy or in combination.^1^

Regardless of the indication in NSCLC, the recommended dosage of pembrolizumab in Canada is 2 mg/kg (maximum 200 mg) intravenously (IV) every 3 weeks (Q3W).^2^ However, in New Zealand and some European countries, the administration of pembrolizumab every 6 weeks (Q6W) at 4 mg/kg (maximum 400 mg) is also authorized.^3,4^ This approval is based on pharmacokinetic models which have demonstrated that several dosages and administration schedules lead to similar exposures. This supports the idea that the dose-response relationship with immunotherapy is not as direct as with chemotherapy.^5^ The survival benefits and adverse effects of this treatment at 2 mg/kg IV Q3W are well documented in the literature.^6-9^ In the 5-year analysis of KEYNOTE-024, a trial of pembrolizumab monotherapy for metastatic NSCLC with PD-L1 TPS ≥ 50%, median overall survival (OS) was 26.3 months and median progression-free survival (PFS) was 7.7 months. Immune-mediated adverse events (AEs) occurred in 34% of patients, including grade ≥ 3 toxicity in 14% of patients.^6^ Median OS of patients who received pembrolizumab in combination with chemotherapy was 22.0 months in metastatic nonsquamous NSCLC in the updated analysis of KEYNOTE-189 and 17.1 months in metastatic squamous NSCLC in the final analysis of KEYNOTE-407. Median PFS was, respectively, 9.0 and 8.0 months. Immune-mediated AEs were reported in 26% of patients (11% of grade ≥ 3 events) in KEYNOTE-189 and in 35% of patients (13% of grade ≥ 3 events) in KEYNOTE-407.^7,8^

At our center, in the context the COVID-19 pandemic, the protocol for the administration of pembrolizumab monotherapy has been revised to a dose of 4 mg/kg IV Q6W in order to reduce visits to the oncology clinic. We conducted this monocentric retrospective study to assess the toxicity and efficacy of a 6 weekly dosing by comparing with data from a cohort of patients previously treated with a 3 weekly regimen also at our center. Data from KEYNOTE-555 cohort B suggest consistent benefit-risk profile and similar overall response rate (ORR), PFS, and AEs in a pembrolizumab 400 mg Q6W dosing regimen compared to a 200 mg Q3W dosing in advanced melanoma.^10^ A recent retrospective study showed that there was no OS difference in patients with stage IV NSCLC and PD-L1 TPS ≥ 50% who were treated with first-line pembrolizumab and dosed at Q3W or Q6W intervals, based on a 2:1 case-matched analysis. The median OS for Q6W dosing was 17.1 months compared to 15.1 months for Q3W dosing, with an HR 0.83 (95% CI 0.49-1.42, *P* = .50).^11^ However, there were only 41 patients in the Q6W cohort and the safety profile of these 2 regimens was not assessed. A study of 45 patients with advanced NSCLC, in whom the pembrolizumab dosage was switched from 200 mg Q3W to 400 mg Q6W, raised concern about new or worsening immune-related AEs, especially pneumonitis in 11 (24%) patients.^12^ To our knowledge, there is no direct comparison in the literature between these 2 regimens for toxicity and efficacy in NSCLC.

## Materials and Methods

### Study Design

This retrospective study included all patients with advanced NSCLC who received pembrolizumab monotherapy 4 mg/kg IV Q6W between March 1, 2020 and December 31, 2021 at Institut universitaire de cardiologie et de pneumologie de Québec (IUCPQ). In the pembrolizumab Q6W group, patients who previously received pembrolizumab Q3W as monotherapy or in combination with chemotherapy were excluded. In order to have a comparative group with an equivalent number of patients, all patients with advanced NSCLC who received pembrolizumab monotherapy 2 mg/kg IV Q3W between January 1, 2017 and January 15, 2019 at our institution were included in the Q3W group. Patients were identified from the Oncology Database (SICTO).

### Data Collection

Data were collected until December 15, 2022. Demographic data collected for this study included age, gender, weight, smoking status, and Eastern Cooperative Oncology Group performance status (ECOG PS). The medical charts were also reviewed for histopathological diagnosis, date of diagnosis, sites of metastases, *EGFR*, *ALK, BRAF, ROS1*, and PD-L1 status. We reported these molecular alterations since they were specifically tested at our institution at this time as we were not routinely performing next-generation sequencing yet. PD-L1 immunohistochemistry (IHC) 22C3 pharmDx was used to determine PD-L1 TPS. Information about treatment and outcomes were collected: line of therapy, treatment start date, number of cycles received, treatment end date, date of disease progression, subsequent lines of therapy, and date of last follow-up or death. Immune-related AEs were recorded until December 15, 2022 for both groups. AEs were graded using the Common Terminology Criteria for Adverse Events (CTCAE) version 5.0. If AEs were not graded in real-time, we determined it according to the clinical evaluation described in the file, biological results or treatment required. Consideration was given to the number of cycles of pembrolizumab at the time of AEs, the management of immune toxicities and their impact on pembrolizumab treatment (continuation, interruption or discontinuation).

The primary outcomes of this study were to compare OS, PFS as well as the occurrence and severity of immune-mediated AEs in patients with advanced NSCLC who received pembrolizumab Q6W vs Q3W. A secondary outcome was to determine the impact of toxicities on continuation of immunotherapy in each group.

### Statistical Analysis

Patient demographics and clinical characteristics were summarized using descriptive methods. For OS and PFS analyses, the Nelson-Aalen estimates of the cumulative hazards were calculated. To compare patients who received pembrolizumab Q6W vs Q3W, the Cox proportional hazard regression analysis was performed to model death-free or event-free follow-up. The adequacy of proportional hazards assumption was checked using a graphical representation of logarithm cumulative hazard rates versus time. A multivariate Cox proportional hazard model was used to compare patients who received pembrolizumab Q6W vs Q3W adjusted for age, gender, stage, PD-L1 TPS, ECOG PS, and histology. All parameters were investigated first using univariate regression models. An artificially time-dependent covariate was added to the model to test the proportionality assumption with covariates. For all variables, the proportional hazards assumptions were not rejected as local tests linked to the time-dependent covariates were not significant. Statistical significance was present with a 2-tailed *P*–value < .05. Analyses were performed using SAS version 9.4 (SAS Institute Inc., Cary, NC).

## Results

### Patients

**Figure 1.**
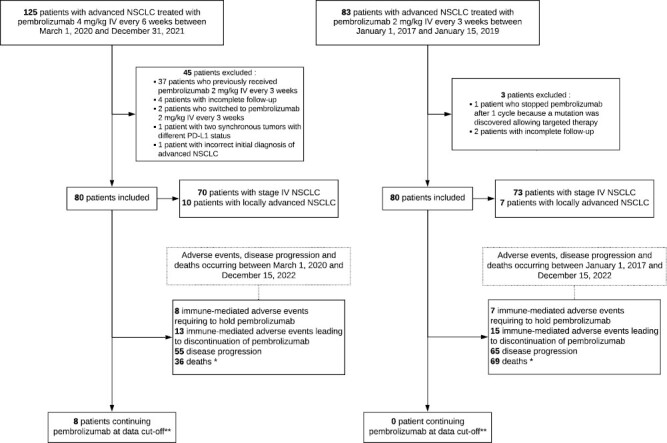
CONSORT diagram. NSCLC = non-small cell lung cancer; PD-L1 = programmed death-ligand 1. *Patient can be in more than one category. **Data cutoff = December 15, 2022.

One hundred and twenty-five patients with advanced NSCLC received pembrolizumab 4 mg/kg IV Q6W between March 1, 2020 and December 31, 2021. Forty-five patients were excluded, of which 37 patients who previously received pembrolizumab Q3W and 2 patients who switched to pembrolizumab Q3W. Four patients had incomplete follow-up, one patient had synchronous tumors with different PD-L1 TPS and the other was initially misdiagnosed because he had concurrent tonsil cancer which was later found. Eighty patients were therefore included in the pembrolizumab Q6W group. Of those, 70 patients had stage IV NSCLC and 10 patients had locally advanced NSCLC but were not candidate for surgical resection or definitive chemoradiation. Between January 1, 2017 and January 15, 2019, 83 patients were treated with pembrolizumab 2 mg/kg IV Q3W. Immunotherapy was discontinued after one cycle because an *ROS1* mutation was discovered allowing targeted therapy in one patient, who was excluded. Two patients had incomplete follow-up. Finally, the pembrolizumab Q3W group was composed of 80 patients, including 73 patients with stage IV NSCLC and 7 patients with locally advanced NSCLC not eligible for curative treatment. The data cutoff date was December 15, 2022 (Fig. 1). Median follow-up was 14.5 ± 8.6 months in the pembrolizumab Q6W group and 18.3 ± 19.6 months in the pembrolizumab Q3W group.

Baseline characteristics of all patients are summarized in Table 1. Most patients in both groups were female, former or current smokers, had adenocarcinoma and were ECOG PS 0-1. Patients in the pembrolizumab Q6W group were slightly older (70.0 vs 66.0 years). There was one patient with an *EGFR* mutation in the Q3W group, while no patient carried a *BRAF V600E* mutation nor an *ALK* or *ROS1* rearrangement. *BRAF V600E* mutation and *ROS1* rearrangements were often unknown because they were not tested systematically especially in the cohort of patients who received pembrolizumab between January 1, 2017 and January 15, 2019. Pembrolizumab was prescribed mainly as first-line monotherapy in patients with PD-L1 TPS ≥ 50%, which represents 100% of patients in the pembrolizumab Q6W group and 91% of those in the pembrolizumab Q3W group. Bone, lung and pleura were the most common sites of metastases.

**Table 1.** Patient characteristics.

	Pembrolizumab Q6W	Pembrolizumab Q3W	*P*-value
	*N* = 80	*N* = 80	
Median age, years ± SD	70.0 ± 7.7	66.0 ± 7.9	.0008
Male sex	35 (44%)	32 (40%)	.7487
Median weight, kg ± SD	65.8 ± 15.3	71.4 ± 14.5	.1637
Former or current smoker	79 (99%)	76 (95%)	.3671
ECOG PS			
0-1	61 (76%)	62 (77%)	.6838
2	15 (19%)	12 (15%)	
3	0	2 (3%)	
Not reported	4 (5%)	4 (5%)	
Histology			
Adenocarcinoma	61 (76%)	61 (76%)	.6887
Squamous carcinoma	12 (15%)	10 (12%)	
Adenosquamous carcinoma	1 (1%)	2 (3%)	
NSCLC NOS	6 (8%)	4 (5%)	
Large cell carcinoma	0	1 (1%)	
Sarcomatoid/pleomorphic carcinoma	0	2 (3%)	
*EGFR* mutation			
Positive	0	1 (1%)	.6530
Negative	67 (84%)	69 (87%)	
Not tested (squamous carcinoma)	12 (15%)	10 (12%)	
Unknown	1 (1%)	0	
*ALK* rearrangement			
Positive	0	0	.6530
Negative	67 (84%)	70 (88%)	
Not tested (squamous carcinoma)	12 (15%)	10 (12%)	
Unknown	1 (1%)	0	
*BRAF V600E* mutation			
Positive	0	0	<.0001
Negative	52 (65%)	7 (9%)	
Not tested (squamous carcinoma)	12 (15%)	10 (12%)	
Unknown	16 (20%)	63 (79%)	
*ROS1* rearrangement			
Positive	0	0	<.0001
Negative	66 (82%)	7 (9%)	
Not tested (squamous carcinoma)	12 (15%)	10 (12%)	
Unknown	2 (3%)	63 (79%)	
PD-L1 status			
<1%	0	0	.0136
1-49%	0	7 (9%)	
≥50%	80 (100%)	73 (91%)	
Sites of metastases at diagnosis			
CNS	12 (15%)	15 (19%)	.6735
Lung	24 (30%)	29 (36%)	.5019
Pleura	23 (29%)	24 (30%)	1.0000
Liver	8 (10%)	10 (12%)	.8032
Bone	21 (26%)	26 (33%)	.4877
Adrenal	13 (16%)	13 (16%)	1.0000
Pericardium	2 (3%)	3 (4%)	1.0000
Other site^*^	10 (12%)	23 (29%)	.0182

^*^The other sites were predominantly intra-abdominal and soft-tissue metastases and extra-thoracic lymphadenopathy.

Abbreviations: ALK: anaplastic lymphoma kinase; CNS: central nervous system; ECOG PS: Eastern Cooperative Oncology Group performance status; EGFR: epidermal growth factor receptor; NOS: not otherwise specified; NSCLC: non-small cell lung cancer; PD-L1: programmed death-ligand 1; Q3W: every 3 weeks; Q6W: every 6 weeks; SD: standard deviation.

### Efficacy

Patients treated with the Q6W dosing regimen received a median number of 8 cycles of pembrolizumab. We considered that a 4 mg/kg Q6W dose corresponded to 2 cycles for calculation. Between March 1, 2020 and December 15, 2022, 55 (69%) patients had disease progression, among which 33/55 (60%) received subsequent lines of treatment, and 36 (45%) patients died. The subsequent therapeutic strategy was chemotherapy in 32 (58%) patients, immunotherapy rechallenge in 1 (2%) patient and targeted therapy in 4 (7%) patients. Five (9%) patients received local treatment by surgery or stereotactic body radiation therapy (SBRT) in the context of oligoprogression. At data cutoff, 5 (6%) patients had completed 2 years of treatment and 8 (10%) patients were still treated with this regimen.

In the pembrolizumab Q3W group, the median number of cycles received was 6. Six (8%) patients had completed 2 years of treatment. Between January 1, 2017 and December 15, 2022, there were 65 (81%) disease progression, among which 35/65 (54%) received subsequent lines of treatment, and 69 (86%) deaths. The subsequent therapeutic strategy was chemotherapy in 33 (51%) patients and immunotherapy rechallenge in 2 (3%) patients. Four (6%) patients received local treatment by surgery or SBRT in the context of oligoprogression (Table 2).

**Table 2.** Treatment and outcomes.

	Pembrolizumab Q6W	Pembrolizumab Q3W
	*N* = 80	*N* = 80
Median dosage of pembrolizumab, mg ± SD	263.2 ± 61.2	142.8 ± 29.0
Median number of pembrolizumab cycles^†^	8	6
Median follow-up, months ± SD	14.5 ± 8.6	18.3 ± 19.6
Median time to onset of adverse effects, months ± SD	4.1 ± 4.2	4.2 ± 6.0
Immune-mediated adverse events requiring to hold pembrolizumab, *n* (%)	8 (10%)	7 (9%)
Immune-mediated adverse events leading to discontinuation of pembrolizumab, *n* (%)	13 (16%)	15 (19%)
Disease progression, *n* (%)	55 (69%)	65 (81%)
Completed treatment, *n* (%)	5 (6%)	6 (8%)
Subsequent lines of treatment in patients with disease progression, *n* (%)		
0	22/55 (40%)	30/65 (46%)
1	19/55 (34%)	19/65 (29%)
2	7/55 (13%)	12/65 (19%)
3	7/55 (13%)	4/65 (6%)
Death, *n* (%)	36 (45%)	69 (86%)
Ongoing treatment with pembrolizumab at data cutoff, *n* (%)^*^	8 (10%)	0

^†^Dose of 4 mg/kg = 2 cycles.

^*^Data cutoff = December 15, 2022.

Abbreviations: NSCLC: non-small cell lung cancer; Q3W: every 3 weeks; Q6W: every 6 weeks; SD: standard deviation.

**Figure 2.**
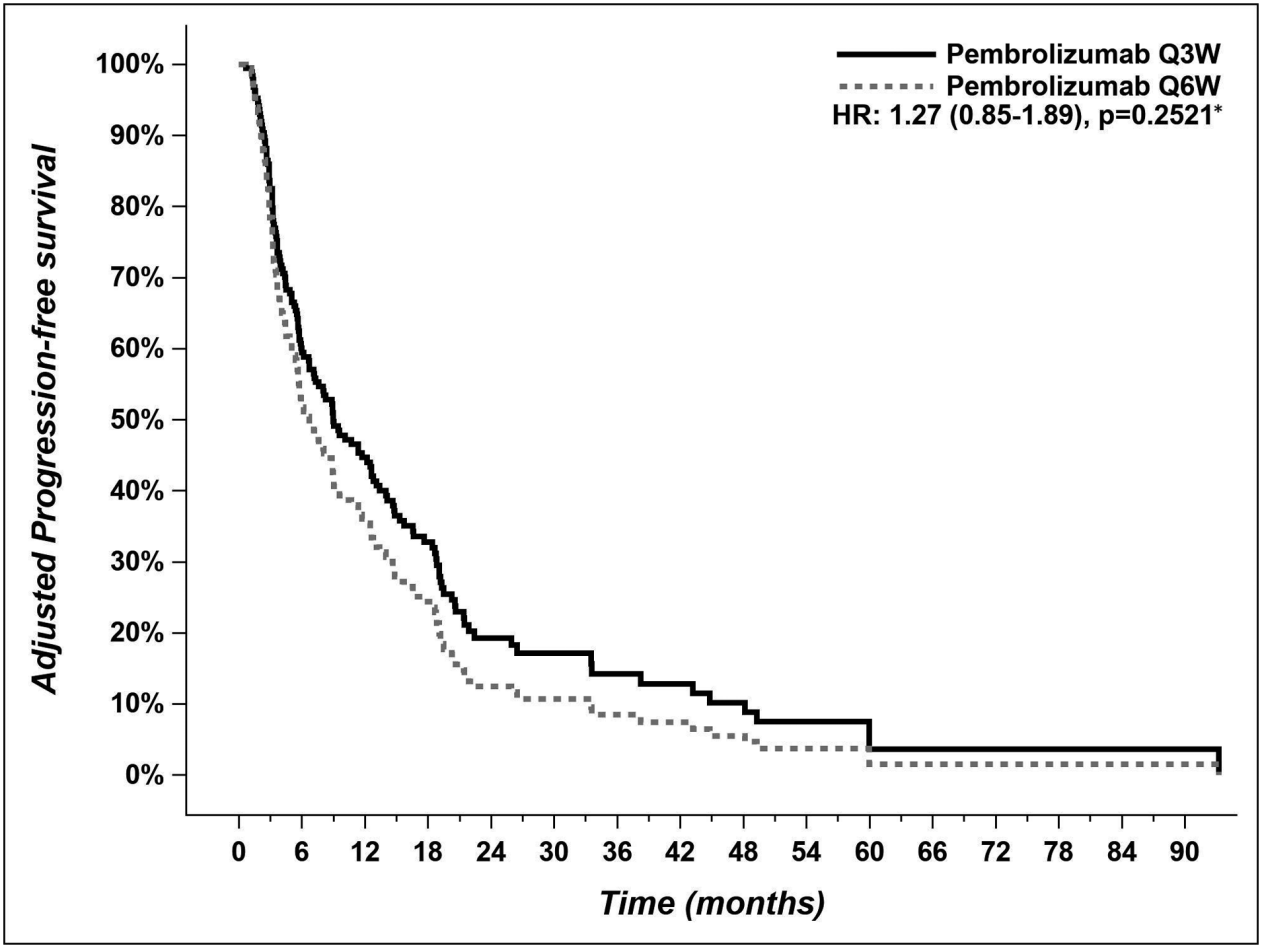
Adjusted progression-free survival of patients who received pembrolizumab Q6W versus patients who received pembrolizumab Q3W. Q3W = every 3 weeks; Q6W = every 6 weeks. *Pembrolizumab Q3W = reference group for HR calculation.

**Figure 3.**
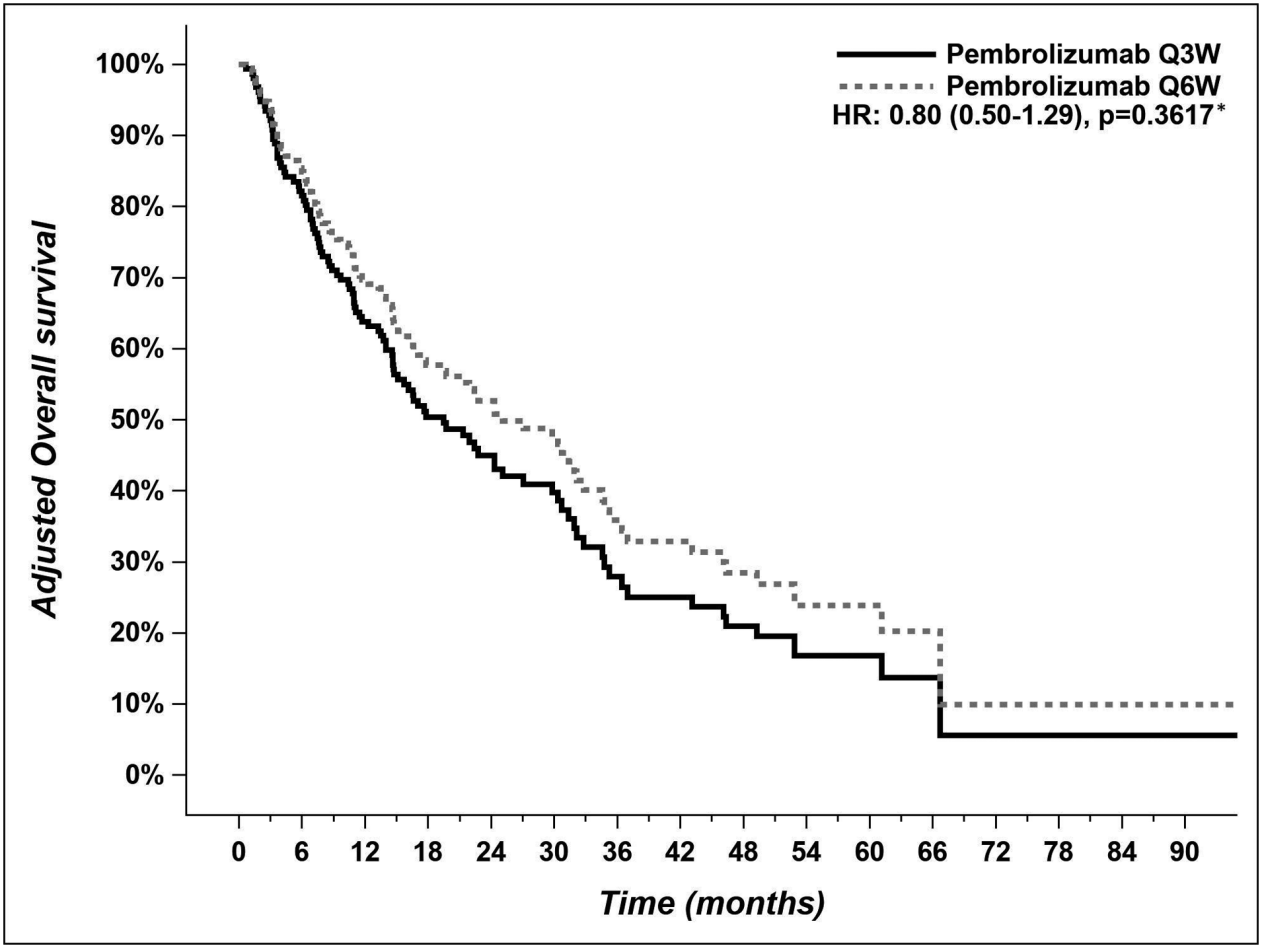
Adjusted overall survival of patients who received pembrolizumab Q6W versus patients who received pembrolizumab Q3W. Q3W = every 3 weeks; Q6W = every 6 weeks. *Pembrolizumab Q3W = reference group for HR calculation.

Median PFS was 6.9 months (CI 95% 5.0-10.7) in the pembrolizumab Q6W group vs 8.9 months (CI 95% 5.6-14.1) in the pembrolizumab Q3W group (Fig. 2, adjusted HR 1.27 (CI 95% 0.85-1.89), *P* = .25). Median OS was not reached in the pembrolizumab Q6W group vs 20.5 months (CI 95% 13.7-29.8) in the Q3W group (Fig. 3, adjusted HR 0.80 (CI 95% 0.50-1.29), *P* = .36).

### Safety

Median time to onset of AEs was 4.1 ± 4.2 and 4.2 ± 6.0 months in the Q6W group and the Q3W grouprespectively. Immune-mediated AEs requiring to hold immunotherapy occurred in 8 (10%) patients with the Q6W regimen and in 7 (9%) patients with the Q3W regimen. There were slightly more immune-mediated AEs leading to discontinuation of pembrolizumab in the Q3W dosing regimen, 15 (19%) patients, compared to 13 (16%) patients in Q6W group (Table 2). Immune-mediated AEs occurred in 76% of patients, including grade ≥ 3 toxicity in 18% of patients in the Q6W group. In the Q3W group, immune-mediated AEs were reported in 74% of patients (19% of grade ≥ 3 events). Immunosuppressive therapy was required for 26 AEs in the Q6W group compared to 23 AEs in the Q3W group. The only fatal event was myocarditis, which happened with the Q3W dosing regimen. There were less pneumonitis with the Q6W regimen, 6 (8%) patients in the Q6W group vs 9 (11%) patients in the Q3W group. Serious pneumonitis (grade ≥ 3) occurred equally in the 2 groups, in 4 (5%) vs 3 (4%) patients in the Q6W group and the Q3W group respectively (Table 3).

**Table 3.** Immune-mediated adverse events.

	Pembrolizumab Q6W *N* = 80						Pembrolizumab Q3W *N* = 80					
	Any grade	Grades 1-2	Grade 3	Grade 4	Grade 5	Grades 3-5	Any grade	Grades 1-2	Grade 3	Grade 4	Grade 5	Grades 3-5
Any immune-mediated adverse event	61 (76%)	47 (59%)	13 (16%)	1 (1%)	0	14 (18%)	59 (74%)	44 (55%)	14 (18%)	0	1 (1%)	15 (19%)
*Skin toxicity*												
Rash	13 (16%)	11 (14%)	2 (3%)	0	0	2 (3%)	15 (19%)	10 (13%)	5 (6%)	0	0	5 (6%)
*Endocrinopathies*												
Hypothyroidism	11 (14%)	11 (14%)	0	0	0	0	7 (9%)	7 (9%)	0	0	0	0
Hyperthyroidism	1 (1%)	1 (1%)	0	0	0	0	1 (1%)	1 (1%)	0	0	0	0
Adrenal insufficiency	3 (4%)	3 (4%)	0	0	0	0	2 (3%)	2 (3%)	0	0	0	0
Diabetes	1 (1%)	0	1 (1%)	0	0	1 (1%)	1 (1%)	0	1 (1%)	0	0	1 (1%)
*Gastrointestinal toxicity*												
Colitis	7 (9%)	5 (6%)	2 (3%)	0	0	2 (3%)	6 (8%)	5 (6%)	1 (1%)	0	0	1 (1%)
Oral mucositis	1 (1%)	1 (1%)	0	0	0	0	0	0	0	0	0	0
Pancreatitis	0	0	0	0	0	0	1 (1%)	0	1 (1%)	0	0	1 (1%)
*Hepatotoxicity*												
Hepatitis	2 (3%)	0	2 (3%)	0	0	2 (3%)	6 (8%)	5 (6%)	1 (1%)	0	0	1 (1%)
*Pulmonary toxicity*												
Pneumonitis	6 (8%)	2 (3%)	4 (5%)	0	0	4 (5%)	9 (11%)	6 (8%)	3 (4%)	0	0	3 (4%)
*Rheumatological toxicity*												
Arthritis	10 (13%)	8 (10%)	2 (3%)	0	0	2 (3%)	8 (10%)	6 (8%)	2 (3%)	0	0	2 (3%)
*Nephrotoxicity*												
Nephritis	3 (4%)	2 (3%)	0	1 (1%)	0	1 (1%)	1 (1%)	1 (1%)	0	0	0	0
*Neurological toxicity*												
Neuropathy	1 (1%)	1 (1%)	0	0	0	0	1 (1%)	1 (1%)	0	0	0	0
*Other toxicity*												
Conjunctivitis	1 (1%)	1 (1%)	0	0	0	0	0	0	0	0	0	0
Xerostomia	1 (1%)	1 (1%)	0	0	0	0	0	0	0	0	0	0
Myocarditis	0	0	0	0	0	0	1 (1%)	0	0	0	1 (1%)	1 (1%)

Abbreviations: NSCLC = non-small cell lung cancer; Q3W = every 3 weeks; Q6W = every 6 weeks.

## Discussion

This single-center retrospective study evaluated the pembrolizumab alternative dosing regimen of 4 mg/kg Q6W compared to a 2 mg/kg Q3W dosing in advanced NSCLC. To decrease the risk of exposure of patients to COVID-19 by reducing visits to oncology clinic, this study was appropriate in this particular context. Furthermore, the Q6W dosing regimen offers quality of life benefits for patients with uncurable lung cancer and decreases health costs to society. The question of the study therefore remains relevant even without the pandemic situation and the study design seems adequate to determine safety and efficacy of the pembrolizumab Q6W regimen.

Regarding patient characteristics, the study sample in each group still represents the usual population of patients with advanced NSCLC. The patients were slightly older, all tumors had PD-L1 status ≥ 50% and there were less commonly metastases in other site in the pembrolizumab Q6W group compared to the Q3W group, but there were no other statistically significant differences between the 2 groups. Most patients were former or current smokers, had adenocarcinoma and plurimetastatic disease. The 2 cohorts had a lower proportion of males compared to that usually observed, 44% and 40% of patients in each group respectively. We do not believe this influenced our results since we used Cox multivariable regression model adjusting for age, gender, stage, PD-L1 TPS, ECOG PS, and histology.

There was no statistically significant difference for PFS and OS between the 2 groups. The shorter follow-up in the Q6W regimen limits the interpretation of the long-term tendency. Median OS was not reached in the Q6W group and was 20.5 months (CI 95% 13.7-29.8) in the Q3W group, which is consistent with studies of first-line pembrolizumab in advanced NSCLC. Median PFS of 6.9 and 8.9 months, respectively, in the Q6W group and the Q3W group were also comparable to data in the literature.

Immune-mediated AEs of grade ≥ 3 were comparable in both groups, 18% of patients in the Q6W group vs 19% of those in the Q3W group, which is consistent with the toxicities data available in the literature. However, our results differ from immune-mediated AEs of any grade described in the studies, mainly related to skin reactions. In our study, all rashes of any grade were considered immune toxicity until proven otherwise. In KEYNOTE-024, KEYNOTE-189, and KEYNOTE-407 trials, only severe skin reactions were considered immune-related AEs and they were comparable in terms of frequency to grade ≥ 3 skin toxicities that occurred in our 2 cohorts of patients.^6-8^ The incidence of pneumonitis of any grade and grade ≥ 3 is similar in the 2 groups of our study and consistent with the data from KEYNOTE-024 where 8.4% of patients had pneumonitis of any grade and 3.2% of patients had grade ≥ 3 pneumonitis.^6^ Between the 2 groups, there were slight differences with regard to immune-mediated AEs requiring to hold (more frequent in the Q6W group) and to discontinue immunotherapy (less frequent in the Q6W group). This might be explained by better understanding and management of immune toxicities acquired through the experience of clinicians with immunotherapy in the most contemporary group, namely the patients who received pembrolizumab Q6W.

### Limitations

Our results are limited by the retrospective nature of the study, by a small sample and by the fact that the study is unicentric. The 2 cohorts were not simultaneous and this could interfere with the management and impact of AEs on continuation of immunotherapy in each group as clinicians have gained experience with pembrolizumab over the years. Also, our analysis would have been more robust if the follow-up of the pembrolizumab Q6W had been longer. We are still convinced that the 2 treatment regimens are comparable in terms of OS, PFS and toxicity.

## Conclusion

In this retrospective study evaluating toxicity and efficacy of pembrolizumab every 6 weeks compared to a standard 3 weekly regimen, 80 patients with advanced NSCLC were included for analysis in each group. Median follow-up was 14.5 months in the Q6W group vs 18.3 months in the Q3W group. Median PFS was 6.9 months (CI 95% 5.0-10.7) in the pembrolizumab Q6W group vs 8.9 months (CI 95% 5.6-14.1) in the pembrolizumab Q3W group (adjusted HR 1.27 (CI 95% 0.85-1.89), *P* = .25). Median OS was not reached in the pembrolizumab Q6W group vs 20.5 months (CI 95% 13.7-29.8) in the Q3W group (adjusted HR 0.80 (CI 95% 0.50-1.29), *P* = 36). The differences obtained are not statistically significant, which supports the argument that the 2 treatment regimens are comparable. The outcomes with the Q6W regimen were also comparable to data in the literature with the Q3W regimen. The occurrence and severity of immune-mediated AEs were comparable, with 18% of grade ≥ 3 events in the Q6W group vs 19% in the Q3W group.

In summary, a dosing regimen of 4 mg/kg every 6 weeks demonstrated a risk-benefit profile which justifies the application and continuation of this dosing regimen for pembrolizumab monotherapy in advanced NSCLC. Based on available pharmacokinetic data, we believe that this also applies for countries using fixed dosing rather than weight-based dosing. This represents a very interesting treatment alternative for patients with palliative therapy and a considerable financial benefit to society in decreasing patient visits. Large multicenter studies with long-term follow-up analysis are needed to support our findings.

## Data Availability

The data presented in this study are available on request from the corresponding author.
